# Therapeutic implications of tumor free margins in head and neck squamous cell carcinoma

**DOI:** 10.18632/oncotarget.21035

**Published:** 2017-09-16

**Authors:** Clara Backes, Henning Bier, Andreas Knopf

**Affiliations:** ^1^ Otorhinolaryngology/Head and Neck Surgery, Klinikum rechts der Isar, Technische Universität München, 81675 München, Germany

**Keywords:** R0, tumor free margin, head and neck, adjuvant therapy, surgery

## Abstract

**Objectives:**

The resection status is one of the most important prognostic factors for patients with head and neck squamous cell carcinoma (HNSCC) concerning overall survival (OS) and recurrence free interval (RFI). To assess whether therapy concepts changed depending on different resection margins and extracapsular extension, OS and RFI data were set into clinical context.

**Methods:**

All HNSCC patients who underwent head and neck surgery with/without adjuvant therapy (n=534) were selected over a ten-year period (2001-2011). Clinical parameters and survival data were collected retrospectively and histopathological analysis of tumor free margins and extracapsular extension were done.

**Results:**

Patients with microscopic *in-sano* resection showed mean OS/RFI of 95/96 months. OS/RFI decreased in microscopic *non-in-sano and* macroscopic *non-in-sano* (56/58 and 35/39 months) as well as in unclear resection margins (63/60 months). Patients with extracapsular extension, microscopic *non*-*in-sano* resection as well as patients with *in-sano* resection after follow up resection demonstrated therapy escalation by adjuvant (chemo-) radiation.

**Conclusions:**

Insufficient surgical margins and extracapsular extension are main risks for a reduced overall and recurrence free survival. Although there is no measure to prevent positive extracapsular extension, clear margins at first pass protect patients from adjuvant therapy escalation.

## INTRODUCTION

Head and neck cancers (HNC) constitute the sixth cause of cancer-related deaths worldwide [1]. In fact, most recent epidemiological data estimates 686,000 new cases and 376,000 deaths in 2012 [2]. HNC are regarded not as a single entity but rather as a heterogeneous group of tumor types with the majority of them (90%) corresponding to squamous cell carcinoma (HNSCC). While HNSCC rate decreased, particularly the rate of laryngeal cancer, the rate of oropharyngeal (HPV-related) cancer increased [3]. Changing trends in the incidence of different HNSCC subtypes with a simultaneous decline in tobacco-associated and increase in human papillomavirus (HPV)-mediated carcinomas result in improvement in 5-year overall survival [4]. A multimodal treatment, consisting of surgery, radiation and chemotherapy, has proved to be successful for most cases [5]. However, there are still controversies when establishing specific guidelines for treatment options [5]. Surgical resection, usually gold standard for treating patients with HNSCC, aims to completely resect the tumor and simultaneously preserve the organ, this way keeping the individuals’ quality of life and improving the overall survival rate (OS) at the same time [6]. Each treatment is done according to different factors, including tumor location, staging, the presence of a positive margin, extracapsular extension (ECE) status and the general health issues of the patient [7, 8]. Nevertheless, cured patients may suffer side effects of aggressive procedures and often, these tumors relapse [9, 10].

One of the main risks for local recurrence represents positive resection margin [11]. Unfortunately, there is not yet consensus in the definition of margins size that could be used in a straightforward manner for treatment decisions [12]. This poses a serious problem as imprecise tumor edges may lead to a second resection if the surrounding tissue is not histopathologically tumor-free [10]. Furthermore, ECE status represents another prognostic factor for OS and RFI. Both, positive margin and ECE positivity are standard indications for adjuvant chemoradiotherapy (CRT) [13–15]. However, based on the difficulty to clarify these risk factors and to optimize therapies, the analysis of cases where CRT means overtreatment, still needs to be addressed. The adequate implementation of adjuvant therapies shows high importance due to its impact on long term morbidities for the patients. Late toxicity effects, such as sicca syndrome, dysphagia and pneumonia were already reported in other studies [16, 17].

The current study investigates OS and recurrence-free interval (RFI) in 534 patients, depending different R status (R0 at first pass, R0 by follow up resection, R1, R2, and Rx) and distinguishes CRT therapy escalation by positive ECE status or insufficient tumor free margins.

## RESULTS

### Clinico-pathological characteristics

A total of 534 patients were analysed for disease related data comprising 341 patients who underwent R0 resection at first pass, 55 patients with R0 status by follow up resection,77 patients with R1 resection, seven patients with R2 resection, and 54 patients with Rx status, respectively (Table 1). Mean patient's age ranged from 55 to 63 years without differences between the groups (p = 0.9; Table 1). Differential analysis of patient's gender demonstrated significant differences between the groups (p = 0.009; Table 1). Post-hoc analysis attributed differences between the groups to a higher percentage of women in R2 resection, while no differences could be observed between other groups. Analysis of location at primary tumor site revealed oropharyngeal carcinoma being the most frequent primary tumor site. There were striking differences between the distributions of tumor localization with respect to different R-status that refer to an increased proportion of sinonasal and oropharyngeal carcinoma after Rx resection (p < 0.0001; Table 1). Interestingly, subgroup analysis of T status failed to achieve differences between the groups (p = 0.13; Table 1). While in a substantial proportion of our patients higher T status rather depends on functional aspects and tumor compartmentation than metric parameter (e.g. maximum tumor diameter) at primary tumor site, analysis of maximum tumor diameter and tumor free margins were performed. Maximum tumor diameter ranged from 24 to 25mm without differences between the groups (p = 0.94, Table 2). The minimum tumor free margin was 4mm in R0 resection at first pass and 5mm after follow up resection (p = 0.053; Table 2). While the vast majority of patients with R0 resection at first pass or after follow up resection showed circumferential margins being the smallest, patients with R1 resection showed a significant increase in small deep margins after post-hoc analysis (p = 0.41; Table 2). Significant differences in N status between the groups referred to increased pN2b/N3 status after R2 resection (p = 0.042; Table 1). The vast majority of patients showed M0 status at the time of diagnosis (p = 0.88; Table 1). Analysis of primary tumor grading demonstrated G2/3 differentiated carcinoma in all groups (p = 0.15; Table 1).

**Table 1:** Clinical and histological parameter of the analyzed cohort

	R0 FP	R0 FUR	R1	R2	Rx	p-value
**n**	341	55	77	7	54	
**Age (years)**						0.9
Median	59	55	56	63	58	
Mean ± SD	59±10	58±10	59±10	61±12	59±10	
**Sex, n (%)**						0.009
Male	310 (91)	46 (84)	60 (78)	6 (86)	43 (80)	
Female	31 (9)	9 (16)	17 (22)	1 (14)	11 (20)	
**Location, n (%)**						<0.0001
Sinonasal system	11 (3)	4 (7)	2 (3)	0	11 (20)	
Nasopharynx	0	0	2 (3)	0	0	
Oropharynx	129 (38)	17 (31)	33 (43)	2 (29)	27 (50)	
Hypopharynx	49 (14)	8 (15)	16 (21)	2 (29)	6 (11)	
Larynx	72 (21)	13 (24)	15 (20)	1 (14)	9 (17)	
Oral cavity	80 (24)	13 (24)	9 (12)	2 (29)	1 (2)	
**T stage, n (%)**						0.13
T1	139 (41)	21 (38)	30 (39)	1 (14)	16 (30)	
T2	124 (36)	19 (35)	30 (39)	1 (14)	25 (46)	
T3	41 (12)	13 (24)	6 (8)	3 (43)	7 (13)	
T4	37 (11)	2 (4)	11 (14)	2 (29)	6 (11)	
**N stage, n (%)**						0.042
N0	171 (50)	30 (55)	38 (49)	1 (14)	24 (44)	
N1	59 (17)	7 (13)	2 (3)	1 (14)	7 (13)	
N2a	83 (24)	12 (22)	30 (39)	3 (43)	15 (28)	
N2b	26 (8)	6 (11)	6 (8)	1 (14)	7 (13)	
N3	2 (1)	0	1 (1)	1 (14)	1 (2)	
**M stage, n (%)**						
M0	338 (99)	55 (100)	76 (99)	7 (100)	53 (98)	0.88
M1	3 (1)	0	1 (1)	0	1 (2)	
**Grading, n (%)**						
G1	14 (5)	2 (4)	1 (1)	0	1 (2)	0.15
G2	184 (54)	28 (51)	28 (36)	5 (71)	23 (43)	
G3	135 (40)	23 (42)	47 (61)	2 (29)	30 (55)	
G4	3 (1)	1 (2)	0	0	0	
Gx	3 (1)	1 (2)	1 (1)	0	0	

**Table 2:** Histological metric data of primary tumor and tumor free margin

	R0 FP	R0 FUR	R1	p-value
**n**	341	55	77	
**Largest primary tumor's diameter [mm]**	24±13	25±13	24±15	0.94
**Minimum tumor free margin [mm]**	4±4	5±5		0.053
**Smallest tumor free margin**				0.41
Circumference	160 (47)	28 (51)	27 (35)	
Deep margin	129 (38)	17 (31)	35 (46)	
Both	52 (15)	9 (16)	15 (19)	

### Resection status determines survival and tumor recurrence

**Figure 1:**
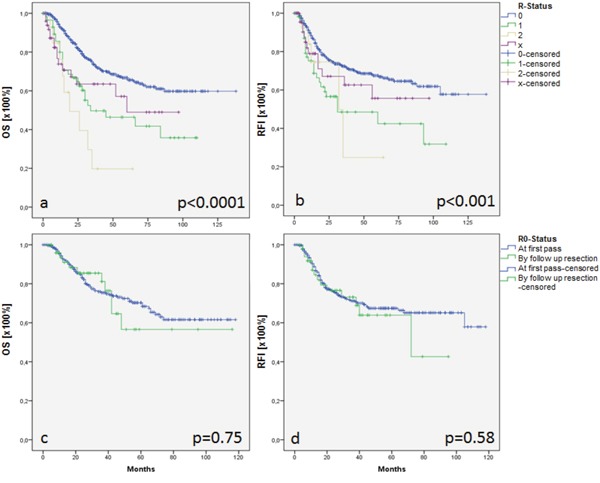
Overall survival (OS) and recurrence free interval (RFI) in patients with R0 resection (**a, b,** blue line), R1 resection (a, b, green line), R2 resection (a, b, yellow line) and Rx resection (a, b, purple line). Subgroup analysis of OS and RFI after R0 resection by first pass (**c, d,** blue line) or follow up resection (c, d, green line).

Analysis of overall (OS) and recurrence free survival (RFI) revealed significant differences between the groups. While patients with R0 resection showed mean OS of 95 months, OS decreased to 56 months after R1 resection and 35 months after R2 resection. Patients who underwent Rx resection showed OS of 63 months (p < 0.0001; Figure 1a). Concordant with results of OS, patients with R0 resection showed prolonged RFI of 96 months when compared with patients after R1 resection (58 months), and R2 resection (39 months), respectively. RFI in patients with Rx resection was 60 months (p < 0.0001; Figure 1b). Forward selected, proportional Cox regression of survival modifying parameters (T, gender, surgical procedure, and localization of primary tumor) identified increasing T status being the only OS-modifying parameter in HNSCC (T1/2 vs. T3/4: HR = 1.6 [95% CI = 1.1-2.3], p = 0.012). No differences could be demonstrated with respect to RFI. Subgroup analysis was performed in patients with R0 resection at first pass and after follow up resection (Figure 1c and 1d). Both, OS and RFI were comparable in patients who underwent R0 resection at first pass and by follow up resection (p = 0.94; Figure 1c; p = 0.36; Figure 1d). Forward selected, proportional Cox regression of OS/RFI modifying parameters (T, gender, surgical procedure, and localization of primary tumor) did not reveal differences between the subgroups.

### Insufficient resection status enforces adjuvant therapy escalation

OS and RFI data were set into clinical context in order to estimate whether treatment regimens changed with respect to different R status. Analysis of surgical approaches showed no differences between surgical concepts at primary tumor site (p = 0.16), while significant differences were demonstrated in the extent of neck dissection (p = 0.007; Table 3). However, post-hoc analysis attributed differences between the groups to a discrepancy of R0 resection at first pass and R1 resection. There were no differences between the other groups. Positive ECE status, being the most important indicator to recommend adjuvant CRT, was demonstrated in 9% of patients after R0 resection at first pass, in 7% of R0 resection by follow up resection, in 9% after R1 resection, 14% after R2 resection, and 15% after Rx resection, respectively (p = 0.61; Table 3). In contrast, adjuvant CRT was applied in 24% and 25% of patients after R0 resection (first pass and follow up resection), in 49% after R1 resection, 100% after R2 resection, and 46% after Rx resection, respectively (p < 0.0001; Table 3). Significant differences in adjuvant treatment concepts were further analysed to assess therapy escalation due to insufficient R status. Therapy escalation by RT/CRT was performed in 10% of patients with R0 resection at first pass due to small (≤ 5mm) tumor free margins. RT/CRT therapy escalation demonstrated significant increase in patients with R0 status after follow up resection (19%), R1 (31%), and Rx status (26%) (p = 0.002; Table 3).

**Table 3:** Therapeutic regimen

	R0 FP	R0 FUR	R1	R2	Rx	p-value
**n**	341	55	77	7	54	
**Surgery at primary tumor site, n (%)**						0.16
Oral and oropharyngeal resection	123 (36)	21 (38)	32 (42)	3 (43)	13 (24)	
Transmandibular resection	37 (11)	4 (7)	9 (12)	0	4 (7)	
Pharyngotomy	28 (8)	1 (2)	0	0	4 (7)	
Vertical partial laryngectomy	3 (1)	2 (4)	4 (5)	2 (29)	0	
Horizontal partial laryngectomy	3 (1)	1 (2)	1 (1)	0	0	
Transoral laser partial laryngectomy	55 (16)	13 (24)	14 (18)	2 (29)	14 (26)	
Pharyngo-/Laryngectomy	57 (17)	7 (13)	11 (14)	0	3 (6)	
Transfacial resection	11 (3)	4 (7)	3 (4)	0	11 (15)	
Other technique	7 (2)	1 (2)	1 (1)	0	4 (7)	
Partial mandibulectomy	17 (5)	1 (2)	2 (3)	0	1 (2)	
**Neck dissection, n (%)**		1 (2)				0.007
None	59 (17)	20 (37)	22 (29)	1 (14)	13 (24)	
Ipsi-lateral	105 (31)	29 (54)	34 (40)	4 (57)	19 (35)	
Bilateral	177 (52)	6 (11)	21 (27)	2 (14)	22 (41)	
**ECE status, n (%)**						0.61
Negative	309 (91)	51 (93)	70 (91)	6 (86)	46 (85)	
Positive	32 (9)	4 (7)	7 (9)	1 (14)	8 (15)	
**Adjuvant therapy, n (%)**						<0.0001
OP only	105 (31)	21 (38)	15 (20)	0	12 (22)	
OP + RT	154 (45)	20 (36)	24 (31)	0	17 (32)	
OP + CRT	82 (24)	14 (25)	38 (49)	7 (100)	25 (46)	
**Adjuvant therapy escalation by insufficient R-status, n (%)**						0.002
None	295 (87)	45 (81)	53 (69)	0	40 (74)	
RT	11 (3)	3 (6)	3 (4)	0	3 (6)	
CRT	35 (7)	7 (13)	21 (27)	7 (100)	11 (20)	

## DISCUSSION

Advanced HNSCC still presents a 5-year rate of disease free survival of less than 50% [18]. Main reason is the difficulty of adequate tumor control when resection margin or ECE status is positive [19, 20]. The current study investigated these factors and their impact on OS, RFI and CRT escalation in 534 patients between the years 2001 and 2011. There was a special focus on adjuvant therapeutic approaches after R0 resection at first pass and by follow up resection in order to set different R status into daily oncological context.

In our series, there were no significant differences in gender and age within each respective R status (R0 at first pass or by followed up resection, R1, R2 and Rx). However, post-hoc analysis constitutes a higher rate of female patients in R2 resection. Teutsch et al. demonstrated similar results, with more challenging surgical access to specific regions in the female cranio-cervical anatomy [21]. The distribution of tumor localization according to R status showed a disproportionately high percentage of sinonasal (20%) and oropharyngeal (50%) carcinomas with Rx resection. Limited possibility for en-bloc resection in these anatomically intricate regions could explain the Rx trends we observe in sinonasal neoplasms [22]. In these cases, a piecemeal surgical intervention is required [22]. The Rx status of oropharyngeal tumors undergoes similar classification. Although there is a variety of surgical methods to achieve a resection of these kind of malignancies, the accessibility to certain sites still needs to be improved [23]. No T status discrepancy classified by maximum tumor diameter was found between the subgroups. However, the post-hoc analysis of the smallest tumor free margin within each R status only revealed significance with R1 resection patients, where the amount of small deep margins increased.

Main focus of our study was to review OS and RFI of our patients depending on tumor's resection margin. As expected, patients with R0 resection showed the highest survival rate with a mean OS of 95 months. After R1 and R2 resection OS declined constantly to 56 and 35 months. Interestingly, patients with Rx resection attained a mean OS of 63 months. Comparable results were reported in earlier studies, although most of them concentrated on the exact margin size and its impact on recurrence and survival rate [24–27]. However, all of them showed the correlation between diminishing tumor free margins and shrinking OS. In accordance to the outcome of OS, patients with R0 resection demonstrated a RFI of 96 months, while R1 resection (58 months) and R2 resection (39 months) showed an impressive decrease. At the same time, Rx resection presented a RFI of 60 months. Eldeeb et al. described similar results in their investigation [26]. Their rate of local recurrence was particularly high, when clear surgical margins were less defined [26]. Adjuvant RT represents therapeutic mainstay in locally advanced tumors or even after insufficient R status to increase both, locoregional control and overall survival [28]. While conventional radiation (70–72 Gy over 7–7.5 weeks with 1.8–2.0 Gy daily) was the treatment of choice until late 1970s, today's altered fractionation significantly increased locoregional control [28]. Since the earliest description by Bennett et al. in 1971 [29], a positive ECE, belongs to the main prognostic factors for OS and RFI. ECE is also most relevant for the indication of CRT [20, 30, 31]. However, a substantial proportion of HNSCC patients undergo adjuvant CRT due to insufficient R status. Concomitant platin-based adjuvant CRT improved outcome in patients with one or both of these risk factors [32]. Accordingly, in our series no differences in positive ECE status were found between the subgroups. Nonetheless, there were a significantly higher number of cases where adjuvant RT/CRT was implemented than cases with positive ECE. Further investigation demonstrated a therapy escalation by aRT/aCRT in 10% of patients with R0 at first pass because of small tumor free margins. Additionally, the adjuvant treatment had a substantial significant increase for R0 after followed up resection (19%), R1 (31%) and Rx (20%). It is important to note that patients with R0 resection at first pass or after followed up resection had no significant difference in OS and RFI in our study. We have to assume that, particularly in the group of patients with R0 status by follow up resection, clear tumor free margins were jeopardized by challenging surgical access of cranio-cervical anatomy that impedes appropriate tissue correspondence. In this scenario, formally achieved R0 status by follow up resection rather refers to Rx or R1 status than R0 status at first pass. Therefore, adjuvant therapy escalation guaranteed survival rates comparable to patients who underwent R0 resection at first pass.

Recent literature estimates that unclear R status effected by the surgeon, leads to an unnecessarily high increase of morbidities for 18% of our patients [15]. The problem of late toxicity is described in earlier studies [16, 17, 23]. In 2012, Keereweer et al. [17] conducted a retrospective study for the morbidity factor in 73 patients after adjuvant treatment. Complications like dysphagia, pneumonia and dehydration were highlighted due to their negative influence on patients´ quality of life [17]. Both, insufficient R status and positive ECE were described in the current study as high risk factors for OS and RFI. Although surgeons cannot influence the outcome of the extranodal extension, they can, through a well-defined cooperation with pathologists, achieve clear tumor free margins [15, 19, 33]. In HNSCC a consensus in definition of clear margins is still missing [19, 34, 35]. Furthermore, literature lacks information about the oncological outcome of patients with tumour free margins at first pass and after follow up resection. Recent meta-analysis in breast cancer indicates better locoregional control after R0 resection at first pass, without relationship between margin widths [36].

## CONCLUSION

Tumor free margins at first pass reduce the necessity of adjuvant therapy escalation and, therefore, acute and late toxicity. Adjuvant therapy escalation in R0 status after follow up resection maintains recurrence-free and overall survival comparable to individuals with R0 status at first pass

## MATERIALS AND METHODS

### Patient selection

All patients who underwent head and neck surgery with/without adjuvant treatment (n=534) in a period of ten years (2001-2011) were included in the current study. Diagnosis of mucosal head and neck squamous cell carcinoma was achieved after histological review by at least two experienced pathologists. Dysplasia, carcinoma *in situ*, and other histologic subtypes such as adenocarcinoma were excluded from the study. Clinical parameters (age, sex, TNM-staging referring to UICC 7^th^ edition, grading, and treatment modalities) and survival data (recurrence, and death/loss to follow-up) were retrospectively collected. The median and mean follow-up time were 24 [11; 45] and 36 months.

### Analysis of tumor free margins and ECE status

Histology was reviewed for maximum tumor diameter, circumferential and deep tumor free margins as well as tumor free margins at first pass or by follow up resection. Lymph node status was classified with respect to UICC 7^th^ edition classification system. ECE status was analysed for all tumor specimens.

### Statistical analysis

Differences between the groups were analyzed using the Chi square test and Fisher exact test for categorical, and the unpaired student's t-test for continuous variables. ANOVA and Tukey's post-hoc was performed for analysis of more than two groups. Survival rates and curves were calculated and illustrated by the Kaplan-Meier method and further analyzed by the log-rank. Variables that revealed prognostic or effect modifying potential on the outcome were subsequently evaluated by the proportional Cox regression for forward selection. p-values <0.05 were considered statistically significant. Statistical analysis was done using SPSS (SPSS Inc., Chicago, IL).
